# Mind the gap: limited knowledge of carbohydrate guidelines for competition in an international cohort of endurance athletes

**DOI:** 10.1017/jns.2023.49

**Published:** 2023-07-03

**Authors:** Gemma Sampson, James P. Morton, Jose L. Areta

**Affiliations:** Research Institute for Sport and Exercise Sciences, Liverpool John Moores University, Tom Reilly Building, Byrom St, Campus, Liverpool L3 3AF, UK

**Keywords:** Carbohydrate, Endurance athlete, Nutrition knowledge questionnaire, Sports nutrition

## Abstract

Despite the well-documented role of carbohydrate (CHO) in promoting endurance exercise performance, endurance athletes typically fail to meet current recommendations in competition. Adequate nutrition knowledge is key to drive athletes’ behaviour, but the current level of knowledge in this population is not known. The present study assessed knowledge of CHO for competition in an international cohort of endurance athletes using the Carbohydrates for Endurance Athletes in Competition Questionnaire (CEAC-Q). The CEAC-Q was completed by 1016 individuals (45 % female), from the United Kingdom (40 %), Australia/New Zealand (22 %), the United States of America/Canada (18 %) and other countries (21 %). Total CEAC-Q scores were 50 ± 20 % (mean ± sd), with no differences in scores between the five subsections (10 ± 5 points, *P* < 0⋅001). Based on typical knowledge and frequency of correct answers, we defined questions with low (0–39 %), moderate (40–69 %) and high (70–100 %) knowledge at a population level. Knowledge deficiencies were identified in questions related to CHO metabolism (Low: 2 out of 5 questions (2/5), Moderate: 3/5), CHO-loading (Low: 2/5, Moderate: 1/5), pre-event CHO (Low: 2/5, Moderate: 2/5), CHO during exercise (Moderate: 4/5) and CHO for recovery (Low: 3/5, Moderate: 1/5). Current CHO amounts recommendations were identified correctly for CHO-loading, pre-competition meal, during competition >2⋅5 h) and post-competition recovery by 28% (Low), 45 % (Moderate), 48 % (Moderate), and 29 % (Low), respectively. Our findings indicate that endurance athletes typically have limited knowledge of carbohydrate guidelines for competition, and we identify specific knowledge gaps that can guide targeted nutrition education to improve knowledge as an initial step towards optimal dietary practice.

## Introduction

Despite a large body of scientific evidence supporting the use of carbohydrates (CHO) to enhance performance of endurance athletes^(1–4)^, a mismatch often exists between current sports nutrition recommendations and CHO intake of athletes in competition^(5–7)^. As an illustrative example, pre-competition CHO intake is recommended to be 8–12 g/kg/d for 24–48 h prior to a prolonged event, however sub-optimal intakes of 2⋅5–7⋅3 g/kg/d for both males and females have been systematically reported on the day prior to competition^(6,8–13)^. Furthermore, just 50 % of triathletes, 30 % of cyclists and 15 % of marathon runners consumed the recommended 60–90 g/h during events lasting >2⋅5 h^(4,14,15)^. These are only examples of the vast reports of athletes systematically failing to achieve what is deemed optimal race nutrition pertaining CHO intake to enhance performance, as outlined in the current guidelines of carbohydrates for competition^(4)^. Given that knowledge is a pre-requisite to make informed decisions^(16)^, it is pertinent to ask if the remarkable and widespread mismatch between current guidelines and intakes of endurance athletes in competition is due to lack of knowledge.

Adequate nutrition knowledge is an essential component to drive an athlete's behaviour and optimise general dietary intake^(16)^. General and sports nutrition knowledge in athletes has previously been assessed using questionnaires with typical scores ranging between 33 and 78 %^(17–19)^, yet little is known about endurance athletes’ knowledge of CHO for competition. Just two published nutrition knowledge questionnaires report CHO knowledge with a distinct subsection score, neither of which are conclusive or ask questions in alignment with current CHO recommendations for endurance athletes^(20,21)^. Despite the importance of CHO for performance during competition, there has been no specific systematic analysis of current knowledge at a population level of endurance athletes’ knowledge of carbohydrate for competition.

To address this, we recently developed and validated a questionnaire to effectively and quickly assess the knowledge of carbohydrates for competition in endurance athletes: the Carbohydrates for Endurance Athletes in Competition Questionnaire (CEAC-Q)^(22)^. During the validation process, we found that a small group of 145 athletes showed total CEAC-Q scores of 46 ± 19 %. We were unable to determine associations between demographic characteristics and knowledge, or specifically characterise in what questions of the questionnaire the athletes’ knowledge is typically lower at a population level. With this in mind, the aim of the present study was to characterise the knowledge of carbohydrates for competition in a large cohort of international endurance athletes using the CEAC-Q with the objectives to (1) determine the relationship between demographic characteristics and knowledge and (2) establish in which specific areas the athlete population is deficient in knowledge.

## Experimental methods

### Study design

The present study assessed the current CHO for competition nutrition knowledge of an international cohort of endurance athletes using the CEAC-Q^(22)^ in English language between August 2019 and June 2020. Briefly, the CEAC-Q consists of twenty-five questions divided into five sections: (1) CHO metabolism, (2) CHO-loading, (3) pre-race CHO meal, (4) CHO during race and (5) CHO for recovery; with each section worth 20 points resulting in a possible total maximum score of 100. The study was advertised online through a variety of social media platforms and the CEAC-Q was completed using SurveyMonkey software (https://www.surveymonkey.com, San Mateo, California, USA), which presented the twenty-five questions in random order after completion of demographic details. Each athlete was allowed to complete the CEAC-Q once, and it was scored as previously described^(22)^.

Individuals were eligible to complete the CEAC-Q for the current study if they were endurance athletes aged >18 years who were actively training and competing in endurance sports events, including cycling, triathlon and running. This study was conducted according to the guidelines laid down in the Declaration of Helsinki and all procedures involving human subjects were approved by the Liverpool John Moores University ethics committee (approval number 19/SPS/025). All participants were provided with the participant information statement, provided written informed consent and electronically agreed to participate.

### Data analysis and statistics

Data means and standard deviations were determined for CEAC-Q total and section scores. Associations between CEAC-Q scores and independent variables were examined using univariate ANOVA and stepwise multiple regression was run to determine which demographic variables predicted total CEAC-Q score. Statistical significance was set at *P* < 0⋅05. Data analyses were conducted in IBM SPSS Version 26 (IBM Corp, Armonk, New York, USA).

We established CEAC-Q reference ranges of ‘Low’ (0–39 %), ‘Moderate’ (40–69 %) and ‘High’ (70–100 %) knowledge based on proximity to *a priori* established values of three groups with increasing levels of knowledge^(22)^ as well the typical marking criteria thresholds used widely in education institutions to determine for fail, adequate and excellent performances. We hypothesized that the ‘moderate’ knowledge band would incorporate the majority of responses and corroborated through *a posteriori* analysis of typical frequency of total CEAC-Q scores in this population (see results).

## Results

### Participant characteristics

A total of 1625 athletes started answering the CEAC-Q, but it was completed by 1016 individuals (attrition rate: 37 %) of mean age 36⋅7 ± 10⋅7 years, from the United Kingdom (*n* 405, 40 %), Australia/New Zealand (*n* 221, 22 %), the United States of America/Canada (*n* 180, 18 %) and other countries (*n* 210, 21 %). Athletes competed primarily in cycling (*n* 400, 39 %), triathlon (*n* 342, 34 %) and running events (*n* 254, 25 %). Other endurance athletes (*n* 20, 2 %) participated in rowing, race-walking, cross-country skiing, obstacle races and open water swimming.

### CEAC-Q total scores are dependent on demographic characteristics

Demographic data collected in relation to gender, age, education, sport, competition level and years competing, whether athletes had worked with a nutritionist or dietitian and their primary source of sports nutrition information and relationship to CEAC-Q scores, is summarised in Table 1. Mean CEAC-Q total scores were 50 ± 20 % and showed clear differences in different demographic characteristics. Stepwise multiple regression identified how an increase in the value of each parameter affected the value of the total CEAC-Q score from a base of 46⋅7 points. In this regard, years competing, competition level, gender, education level, worked with registered sports nutritionist or dietitian, primary source of nutrition information, weight and age resulted in factors of 2⋅37, 0⋅72, −4⋅72, 2⋅17, 3⋅43, 2⋅72, −0⋅12 and −0⋅42, respectively, that significantly predicted CEAC-Q score. However, the predictive power of total CEAC-Q score using demographics within the regression equation [46⋅7 + (2⋅37*years competing) + (0⋅72*competition level) – (4⋅72*gender) + (2⋅17*education level) + (3⋅43*nutritionist) + (2⋅72*source nutrition info) – (0⋅12*weight) – (0⋅42*age)] was poor *F*(8,1003) = 37⋅42, *P* < 0⋅001, *R*^2^ = 0⋅23.

**Table 1.** Participant demographics and corresponding CEAC-Q score per sub-group.

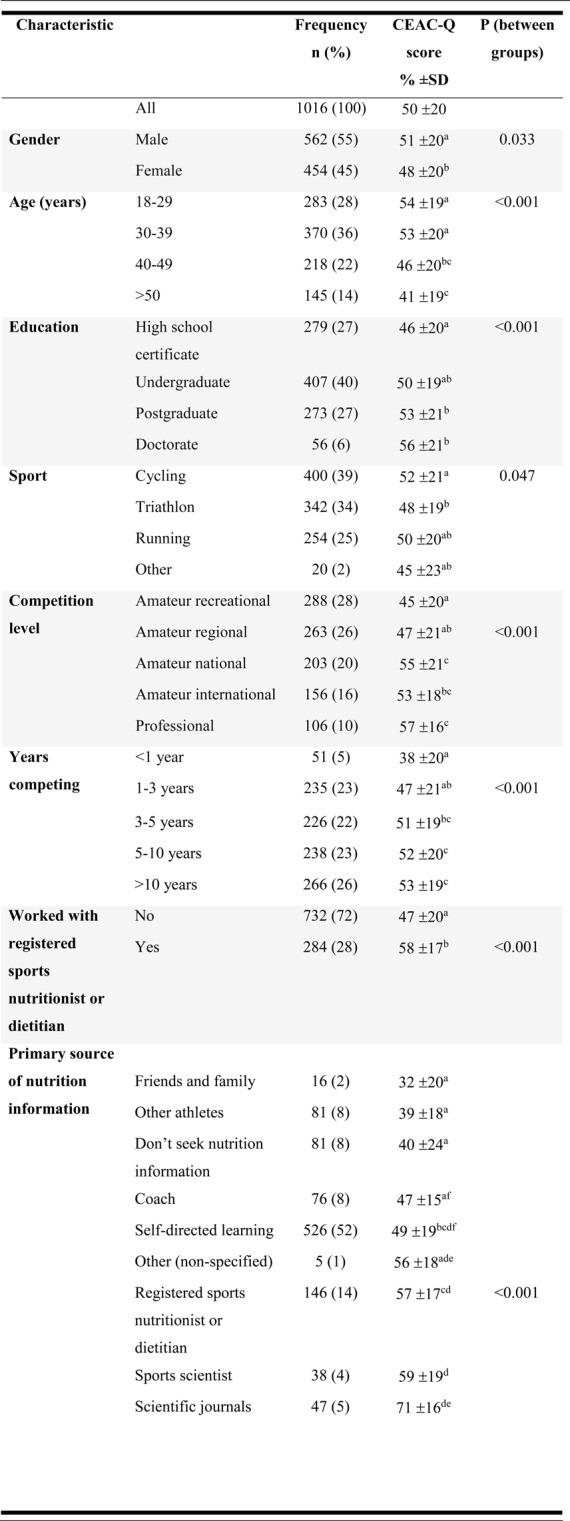

Significant differences of pairwise comparisons for each variable are reported with superscript next to the corresponding values. Any two values that are not followed by the same single letter, are statistically significantly different (*P* < 0.05)

CEAC-Q scores increased incrementally with years of competitive experience, with those competing for less than 1 year scoring significantly less than those who had been competing for over 10 years (38 ± 20 % *v.* 53 ± 19 %, *P* < 0⋅001). CEAC-Q scores increased with competitive level with recreational athletes scoring significantly lower than professional athletes (45 ± 20 % *v.* 57 ± 18 %, *P* < 0⋅001), respectively. Knowledge increased incrementally with education level from high school certificate scoring lowest to doctorate scoring highest (46 ± 20 % *v.* 56 ± 21 %, *P* < 0⋅001). Male athletes scored higher than females (51 ± 20 % *v.* 48 ± 20 %, *P* = 0⋅033) and CEAC-Q scores decreased with age, with athletes aged >50 years scoring lower than those aged 18–29 (41 ± 19 % *v.* 54 ± 19 %, *P* < 0⋅001). Most athletes (*n* 732, 72 %) had never seen a registered sports nutritionist or dietitian, and those who did achieve higher CEAC-Q scores (47 ± 20 % and 58 ± 17 %, respectively, *P* < 0⋅001). Over half the athletes surveyed (*n* 526, 52 %) used self-directed learning including websites, books and podcasts as their primary source of sports nutrition information. CEAC-Q scores increased with education level and those athletes who referred to scientific journals for nutrition education showed the highest scores of all subgroups (71 ± 16 %, *P* < 0⋅001).

### Total knowledge scores and frequency of answers in knowledge bands

**Fig. 1.**
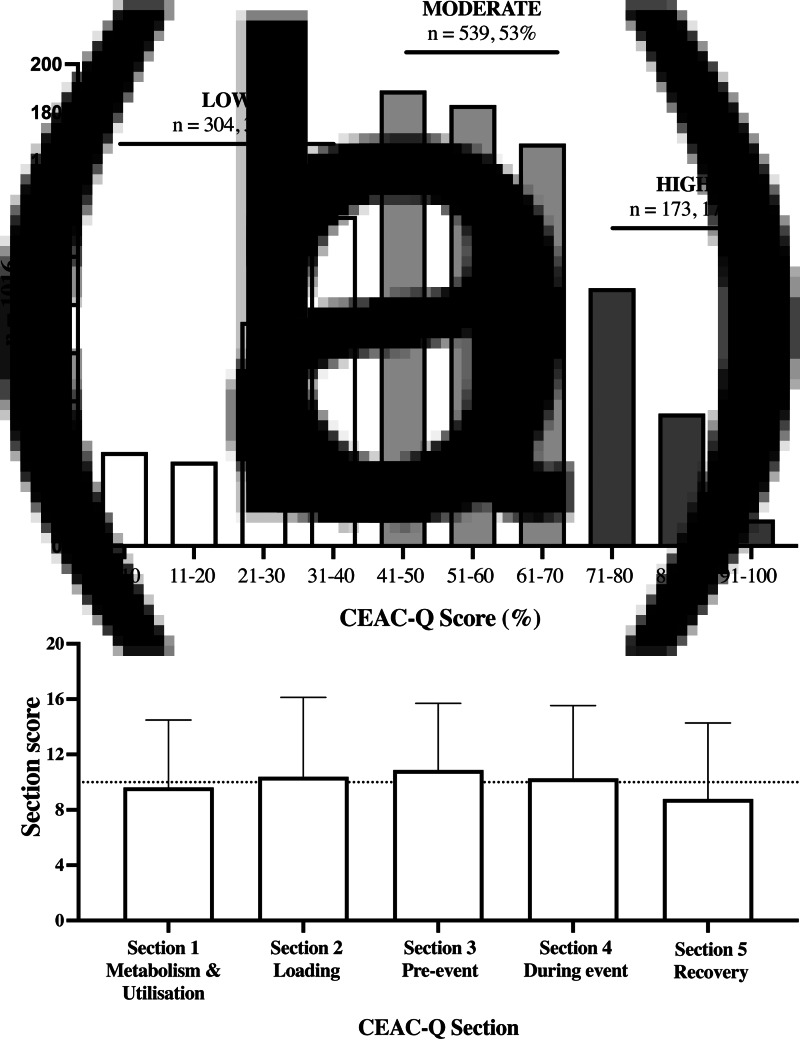
(a) CEAC-Q scores histogram (mean, %) and (b) mean CEAC-Q sections score is represented by horizontal dotted line (mean ± sd)_._

The total knowledge histogram showed a bell-shaped distribution of the population (Fig. 1(a)) and fitted our *a priori* expected frequency in knowledge bands of low, moderate and high levels of knowledge. Based on our *a priori* cut-off values for knowledge bands, the frequency of distribution was highest for moderate knowledge, as expected (41–70 %; *n* 539, 53 %), followed by low knowledge (0–40 %; *n* 304, 30 %) and high knowledge (71–100 %; *n* 173, 17 %). Given the distribution fitted our *a priori* hypothesis and the moderate knowledge band including the majority (>50 %) of the population within 1 standard deviation from the centre of the bell (median 50⋅7 %), reflected the values observed within our validation study consisting of non-athlete general population (17 ± 20 %), endurance athletes (46 +19 %) and sports dietitians and nutritionists (76 ± 10 %, *P* < 0⋅001)^(22)^, and also fitted typical scoring systems used in education systems, we preserved the knowledge level classification system.

### Subsection scores are not different, but there are large differences in knowledge of individual questions in the population

**Fig. 2.**
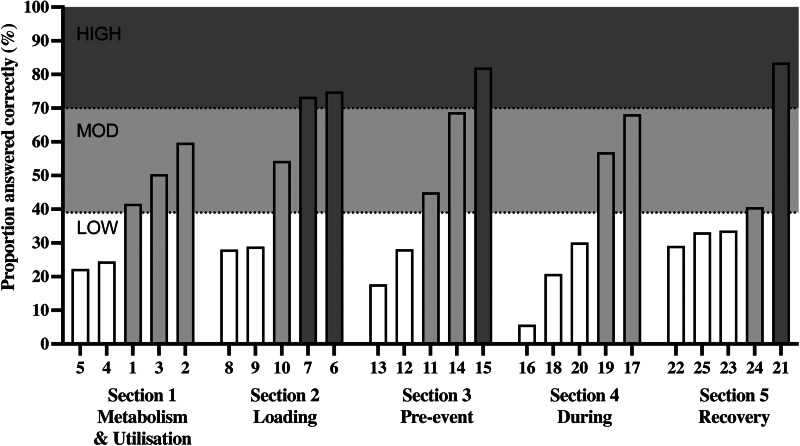
Proportion of correctly answered CEAC-Q questions, grouped and ranked by section. Bar shading represents questions with low (<40 %), moderate (40–69 %) and high (>70 %) knowledge. Numbers in the *x*-axis refer to CEAC-Q question number. Questions 1, 16, 18 and 20 contain multiple answers, final score shown is from when all the answers within that question were correct.

No clear difference in endurance athletes’ overall knowledge of CHO metabolism and utilisation, loading, pre-event meal, during competition or recovery was identified, with average section scores of 10 ± 5 points out of 20 (Fig. 1(b)). However, a detailed analysis of knowledge of each question within sections showed a large variation in the frequency of correct answers for individual questions (Fig. 2, Table 2), ranging from 6 % (Question 16) to 84 % (Question 21).

**Table 2.** CEAC-Q responses for each individual question identify knowledge gaps

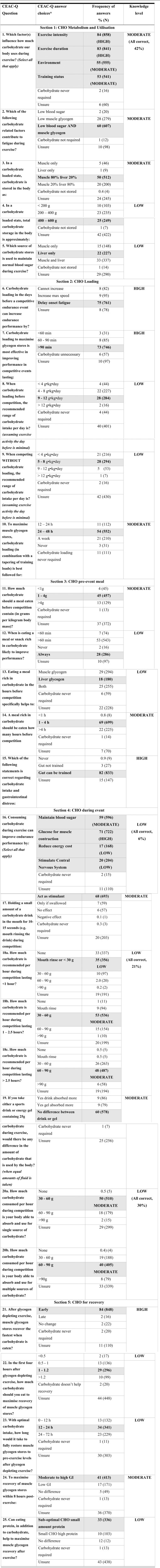

* Contraction of the original answers for practical representation on the table, due to space constraints. Full CEAC-Q questionnaire is available in Sampson *et al.*^(22)^

#### Section 1: Carbohydrate metabolism and utilisation

Limited knowledge of CHO metabolism and utilisation was evidenced by questions 4 and 5 reflecting low knowledge and questions 1, 2 and 3 reflecting moderate knowledge (Fig. 2, Table 2). Specifically, knowledge on total storage of carbohydrates was low (Question 4, 25 % correct), the proportion of distribution of carbohydrates in muscle and liver, moderate (Question 4, 50 % correct) and the role of liver glycogen stores, low (Question 5, 22 % correct). The identification of blood sugar and muscle glycogen as factors related to fatigue reflected moderate knowledge (Question 2, 60 % correct). Overall, the factors that influence carbohydrate use showed a moderate level of knowledge (Question 1, 42 % correct) with the uneven frequency of correct answered sub-questions: knowledge intensity and duration were high (Question 1, 84 and 83 % correct, respectively), but correct identification of environment and training status as contributing factors was only moderate (Question 1, 55 and 53 % correct, respectively).

#### Section 2: Carbohydrate loading

Limited knowledge of CHO-loading was evidenced by questions 8 and 9 reflecting low knowledge and question 10 reflecting moderate knowledge (Fig. 2, Table 2). Specifically, low knowledge was reflected in identification of the amount of carbohydrates required for carbohydrate loading (Question 8, 28 % correct) and for when carbohydrate loading is not needed (Question 9, 28 % correct). The knowledge on the typical timeframe required for carbohydrate loading was moderate (Question 10, 54 % correct).

#### Section 3: Pre-event carbohydrate meal

Limited knowledge of CHO in the pre-event meal was evident through questions 12 and 13 reflecting low knowledge and questions 11 and 14 moderate knowledge (Fig. 2, Table 2). Specifically, the identification of 1–4 g/kg CHO in the meal before competition (Question 11, 45 % correct) and the timing of 1–4 h pre-exercise (Question 14, 69 % correct) showed a moderate level of knowledge. We identified a low level of knowledge of when pre-event carbohydrates are likely to enhance performance (Question 11, 28 % correct) and that the primary reason to consume CHO in the hours before exercise is to replenish liver glycogen stores (Question 13, 18 % correct).

#### Section 4: Carbohydrates during the event

Limited knowledge of CHO during competition was evidenced by questions 16, 18 and 20 reflecting low knowledge and question 17 and 19 reflecting moderate knowledge (Fig. 2, Table 2). Specifically, while 83 % of athletes were able to identify at least 1 of all the mechanisms by which carbohydrates consumed during competition may improve performance, correct identification of all the factors was low (Question 16, 6 % correct). A more granular analysis of the different components contributing towards this question shows that knowledge of the contribution of glucose for muscle contraction knowledge was high (71 % correct), the maintenance of glycaemia reflected moderate knowledge (59 % correct), but stimulation of Central Nervous System (CNS) and reduction of energy cost of exercise reflected low knowledge (20 and 17 % correct, respectively). Overall, the correct identification of all the recommended amounts of CHO depending on event duration reflected low knowledge (Question 18, 21 % correct), also showing divergent in knowledge of the different sub-questions of needs for different durations: CHO intake for events lasting less than 1 h (low, 35 % correct), events duration of 1–2⋅5 h and >2⋅5 h duration (moderate, 53 and 48 % correct, respectively). The effect of CHO form consumed (solid *v.* liquid, Question 19, 60 % correct) or CHO type (single *v*. multiple CHO source, e.g. glucose *v.* glucose and fructose, Question 20, 30 % correct) on CHO utilisation rates reflected moderate and low knowledge, respectively. While half the athletes surveyed (50 %) understood that the body can use approximately 60 g/h of CHO during exercise from a single source, only 40 % knew that this could be increased to 90 g/h when multiple sources of CHO are consumed (Question 20), and just a third understood that with optimal CHO intake glycogen levels could be restored within 12–24 h (Question 23, 34 % correct).

#### Section 5: Carbohydrates for recovery after event

Limited knowledge of CHO recovery nutrition was evidenced by questions 22, 23 and 25 reflecting low knowledge and question 24 reflecting moderate knowledge (Fig. 2, Table 2). While the need of carbohydrate immediately after exercise was identified and reflected high knowledge (Question 21, 84 % correct), the amounts required to maximise recovery of muscle glycogen reflected low knowledge (Question 22, 29 % correct), as well as the time required to replenish muscle glycogen after glycogen-depleting exercise (Question 23, 34 % correct). The selection of moderate to high glycaemic index (GI) carbohydrates to maximise recovery of muscle glycogen early after recovery showed moderate knowledge (Question 24, 41 % correct), but the knowledge on co-ingestion of protein to increase muscle glycogen with sub-optimal carbohydrate ingestion was low (Question 25, 33 % correct).

## Discussion

Utilising a large international cohort of endurance athletes, we characterised for the first time the level of knowledge of carbohydrate guidelines for competition at a population level utilising the recently validated CEAC-Q questionnaire^(22)^. We defined levels of low (<40 %), moderate (41–70 %) and high (>70 %) knowledge and observed that demographic characteristics were not strong predictors of knowledge levels. Our current findings of average CEAC-Q scores in athletes of 50 ± 20 %, corroborate our validation study reporting 46 ± 19 %^(22)^ and support the idea that the majority of the athletic population has limited knowledge (Fig. 1). The demographics with lesser exposure to quality sources information appears to be more likely to have lower nutrition knowledge and despite there being no differences in knowledge between CEAC-Q section scores (10  ± 5 points out of 20), we found large differences in knowledge of specific questions. These findings allow the CEAC-Q to be used by sports dietitians to guide development of future educational material targeted to bridge the gap between current recommendations of best practice and endurance athletes’ knowledge at a population level.

Our findings expand an existing body of evidence on general sports nutrition knowledge in endurance athletes by specifically investigating CHO-specific knowledge for competition using the CEAC-Q. Mean CEAC-Q scores of 50 % were in alignment with existing nutrition knowledge questionnaires for endurance athletes assessing either general nutrition knowledge^(23–27)^ or sports specific nutrition knowledge^(7,28–33)^ which demonstrate that athletes achieve general and sports nutrition knowledge scores ranging between 33 and 78 %^(17–19)^. Studies previously assessing components of CHO knowledge and practice were not assessed within a single knowledge assessment tool to clearly define knowledge gaps and examine factors influencing knowledge. Our results indicate that, as with general sports nutrition knowledge questionnaires, CHO-specific nutrition knowledge of endurance athletes is limited.

Demographics are typically important in determining the level of nutrition knowledge in athletes^(17)^, and while we show differences in knowledge in different demographics (Table 1), their predictive power was poor and their clinical relevance in some cases debatable, but overall they suggest that access to high-quality information is associated to higher levels of knowledge. Male athletes achieved significantly higher CEAC-Q scores than females, but the difference equates to answering on average an additional 0⋅5 questions correctly. We observed, however, notable increases in CEAC-Q score in athletes competing with more experience and at a higher level, showing an average of 12 points difference, between amateur recreational (45±20 %) and professional athletes (57±16 %, Table 1) which is in contrast to Trakman *et al.*^(29)^ who found no difference in general nutrition knowledge between amateur and professional athletes. Elite athletes are less likely to use social media and online information for nutrition advice^(34)^ and may also have greater access to a sports dietitian to provide them with quality nutrition advice, which may partially explain their greater knowledge. Accordingly, the primary source of nutrition knowledge played a remarkable role in predicting CEAC-Q scores (Table 1) and the largest average difference within any demographic category of 38 points is observed between athletes that rely on knowledge from friends and family (32±20 %) compared with those who seek information from a high-quality information source such as scientific journals (71±16 %). Athletes report being hesitant to receive or seek help with their nutritional choices, instead preferring to rely on their own previous knowledge or self-directed research and social media for advice^(6,29,34,35)^. Indeed, only 3 % of runners and 5 % of cyclists sought professional advice to guide their competition nutrition practices^(6,7)^. Overall, these data suggest that the demographics which are less likely to have exposure to high-quality sources of sports nutrition information are more likely to have lower levels of knowledge, highlighting the importance of promoting access to quality information and educational resources to maximise athletes’ knowledge.

It is also noteworthy that the knowledge differences in the population (Fig. 1(a)) were not arising from differences in knowledge in different sections (Fig. 1(b)), but from differences in knowledge in specific questions (Fig. 2, Table 2). Previous studies indicated low knowledge of current CHO recommendations relevant to competition^(6,7,36)^ where just 4 % of amateur runners correctly identified the amount of CHO required for CHO-loading, and the majority (85 %) selecting ‘*I don't know*’^(6)^. Likewise, just 1 % of amateur runners^(6)^ and 25 % of triathletes^(36)^ correctly identified the recommended amount of CHO to consume post-exercise for optimal recovery and 43 % chose ‘*I don't know*’ or answered incorrectly. Until now, little was known how much endurance athletes know about current CHO recommendations, and whether nutrition education on recommended CHO intakes to be consumed within competition have been well translated, explained and understood by athletes. Using the CEAC-Q, we identified that endurance athletes possess low theoretical knowledge to identify current recommendations for CHO-loading (28 %), or post-competition recovery (29 %) and moderate theoretical knowledge for the pre-intake meal (45 %) or during competition of duration >2⋅5 h (48 %), all of which may be related to poor practice in areas that are particularly relevant for athletes in competition.

With mean CEAC-Q scores of 50 %, our findings indicate that endurance athletes typically have limited knowledge of carbohydrates for competition which appears similar to general sports nutrition knowledge levels. Current CHO recommendations were identified by 28 % athletes for CHO-loading, 45 % for pre-competition meal, 48 % for during competition >2⋅5 h and 29 % for post-competition recovery. CEAC-Q scores suggest that the demographics with less exposure to quality information are likely to have less knowledge and we identified specific knowledge gaps that can guide targeted nutrition education designed to improve knowledge driving optimal dietary practice. A possible limitation of the current study is the use of a single recruitment strategy via social media and inclusion of mainly developed countries. We intended to have the broadest possible reach for target population and believe this may be representative of the endurance athlete population in these countries, but future studies may investigate if different recruitment strategies results in assessment in of different demographics, resulting in different overall levels of knowledge.

Nonetheless, the CEAC-Q can identify gaps in CHO nutrition knowledge of endurance athletes at a population level and current findings can be used to guide development of future nutrition educational material and interventions designed to bridge the gap between current recommendations of CHO best practice, athletes’ knowledge and dietary behaviours within competition. To address population level knowledge gaps (Fig. 2, Table 2), the content of questions 4, 5, 8, 9, 12, 13, 16, 18, 20, 22, 23 and 25 (low knowledge) should be emphasized in future educational material, followed by the knowledge of question 1, 2, 3, 10, 11, 14, 17, 17 and 24 (moderate knowledge). Lesser emphasis is required to enhance the knowledge of questions 6, 7, 15 and 21 as knowledge was high in these. The CEAC-Q demonstrates that endurance athletes have low to moderate levels of knowledge of current CHO guidelines and it is possible that athletes would benefit from quality educational resources (which provide scientifically proven knowledge) targeting these specific questions providing clear and objective information to improve theoretical knowledge.

In summary, the present data highlight crucial gaps in knowledge of optimal CHO for competition which build a growing body of evidence that many athletes continue to be unaware or lack understanding of the performance benefits of CHO and best practice recommendations of how much CHO to consume to optimise performance^(5–7,11)^. While adequate nutrition knowledge may be key driver of behaviour, the relationship between endurance athletes’ theoretical knowledge of CHO recommendations and dietary practice within competitive settings is yet to be determined. Many factors influence food choices^(37,38)^ and increased levels of knowledge via nutrition education in isolation does not guarantee translation into general dietary practices of athletes^(16,39,40)^. Further research understanding these factors and clarifying how knowledge gaps identified by the CEAC-Q relate to dietary practice may help answer why many endurance athletes fail to consume optimal CHO within competition and bridge the gap between current scientific knowledge and population knowledge.
